# Supplementary material for: Maximizing Anticancer Response with MPS1 and CENPE Inhibition Alongside Apoptosis Induction
Source: Pharmaceutics. 2023 Dec 29;16(1):56. doi: 10.3390/pharmaceutics16010056 (PMC10818680; doi:10.3390/pharmaceutics16010056)
Supplement: Supplementary file 1 [file pharmaceutics-16-00056-s001.zip › Supplementary file Pharmaceutics 2712023.pdf]

Supplementary file

(a) GSK923295 and Navitoclax IC_{50} in 2D HPAEpiC

Drugs	IC50 (nM)
GSK923295	> 1,000
Navitoclax	9790 ± 770

(b) BAY1217389 and Navitoclax IC_{50} in 2D HPAEpiC

Drugs	IC50 (nM)
BAY1217389	> 8,000
Navitoclax	9920 ± 760

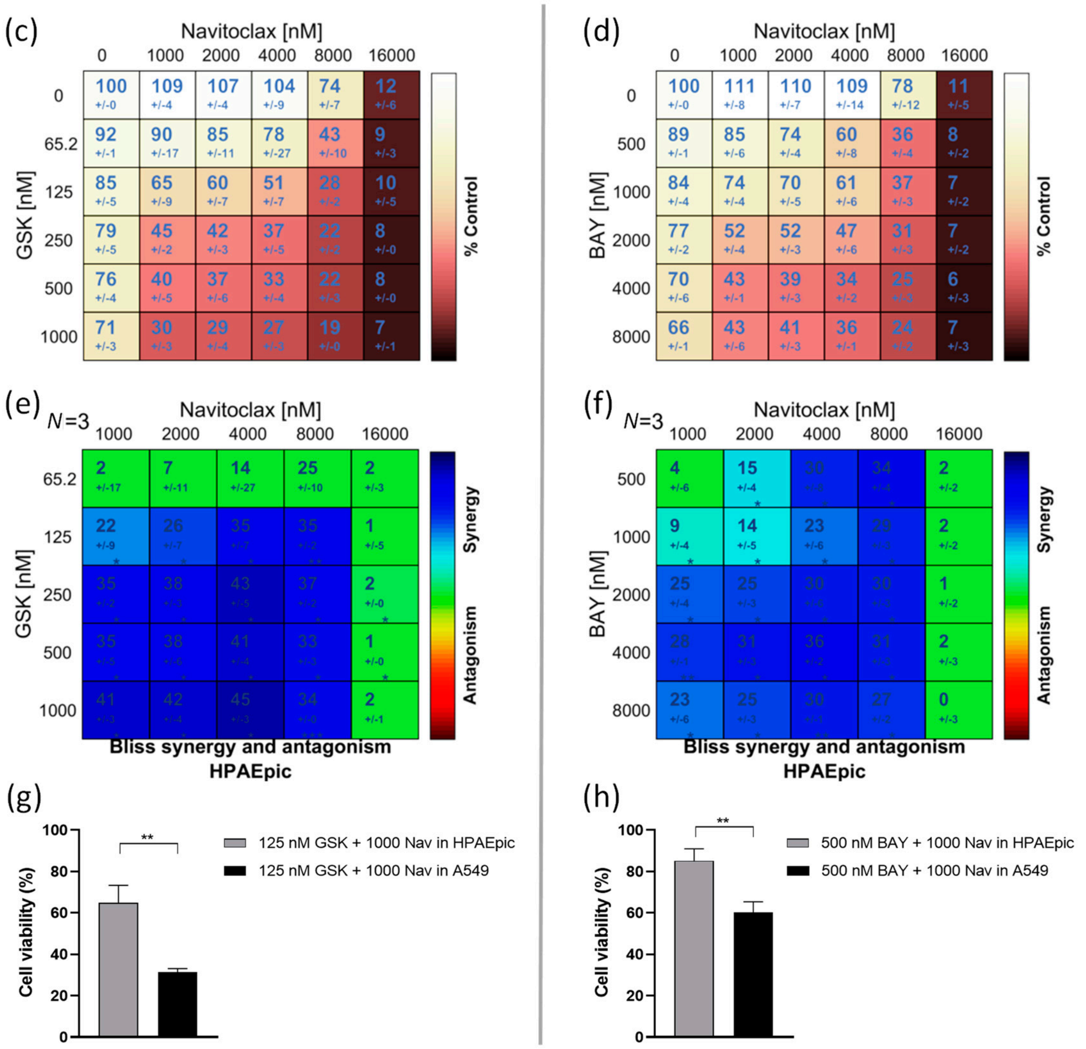

Figure S1: Non-cancer lung cells are less sensitive to GSK923295 + navitoclax and BAY1217389 + navitoclax combinations treatments than lung cancer cells. IC_{50} of all compounds in HPAEpiC cells (a,b). Cell viability (%) of single or combination treatments after 48 hours of drug exposure in HPAEpiC cells (c,d), from 3 independent experiments determined by MTT assay. Synergy scores calculated by the Bliss model of Combenefit software with statistical relevance of * $p < 0.05$, and ** $p < 0.01$. Asterisk indicates synergism effects (e,f). Cell viability (%) of GSK923295 + navitoclax (g) and BAY1217389 + navitoclax (h) at the concentration of the respective synergistic points on A549 and HPAEpiC cells, with statistical relevance of ** $p < 0.01$ by unpaired t test.

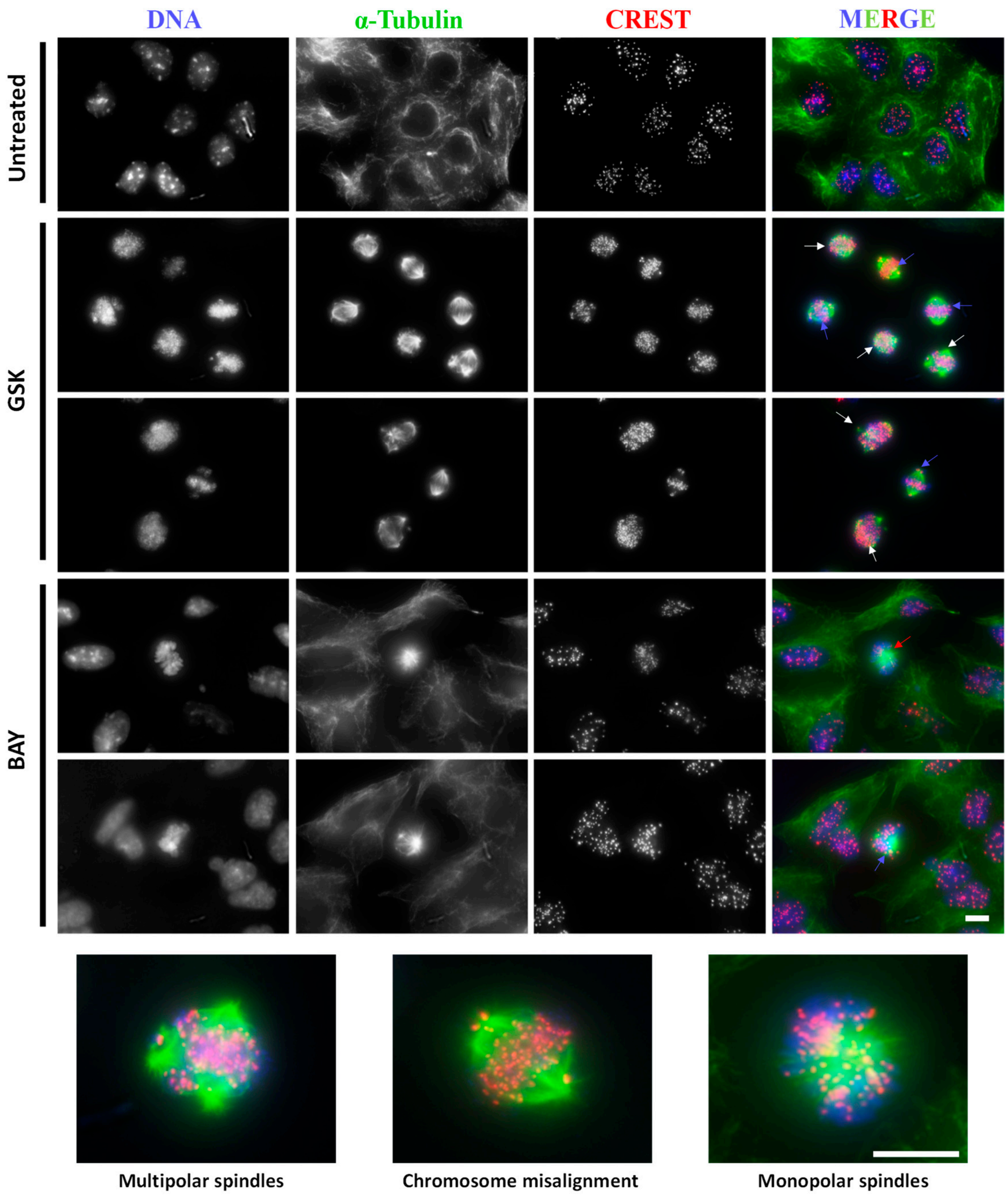

Figure S2: Inhibition of CENPE or MPS1 leads to mitosis abnormalities. Representative immunofluorescence images of A549 cells after 24 hours treatment with 125 nM of GSK923295 or 500 nM of BAY1217389. Cells were immunostained for α-tubulin, to visualize microtubules (green), for CREST (red) to visualize centromeres, and the DNA (blue) was stained with DAPI. Bar, 5 µm. White arrows indicate the presence of multipolar spindles. Blue arrows indicate the presence of chromosome misalignment. Red arrows indicate the presence of monopolar spindles.

(a) GSK923295 and Navitoclax IC_{50} in 2D NCI-H460

Drugs	IC_{50} (nM)
**GSK923295**	160 ± 80
**Navitoclax**	8930 ± 540

(b) BAY1217389 and Navitoclax IC_{50} in 2D NCI-H460

Drugs	IC_{50} (nM)
**BAY1217389**	5500 ± 960
**Navitoclax**	8100 ± 840

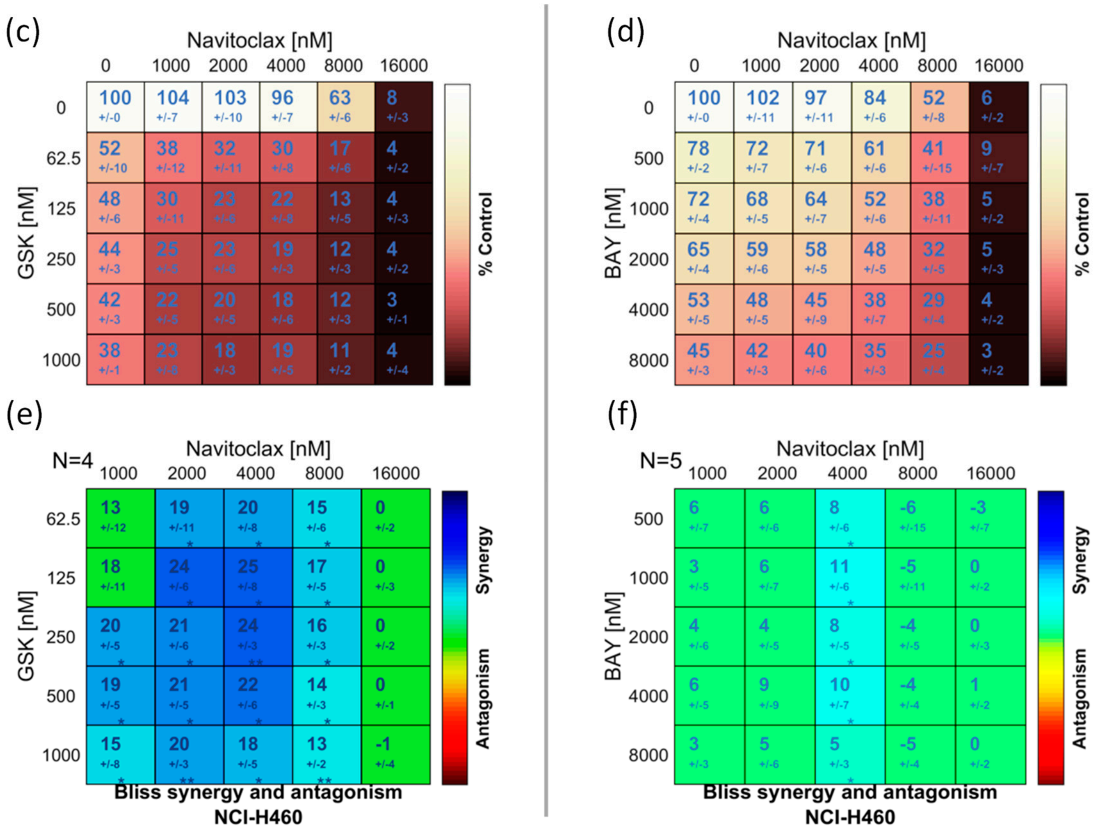

Figure S3: GSK923295 + navitoclax and BAY1217389 + navitoclax combinations potentiate cyto-toxicity in NCI-H460 lung cancer cells. IC_{50} of all compounds in NCI-H460 cells (a,b). Cell viability (%) of single or combination treatments after 48 h of drug exposure in NCI-H460 cells (c,d), from more than 3 independent experiments as determined by MTT assay. Synergy scores calculated by the Bliss model of Combenefit software 2.021 with statistical relevance of * $p < 0.05$, and ** $p < 0.01$ in NCI-H460 cells. Asterisk indicates synergism effects (e,f).

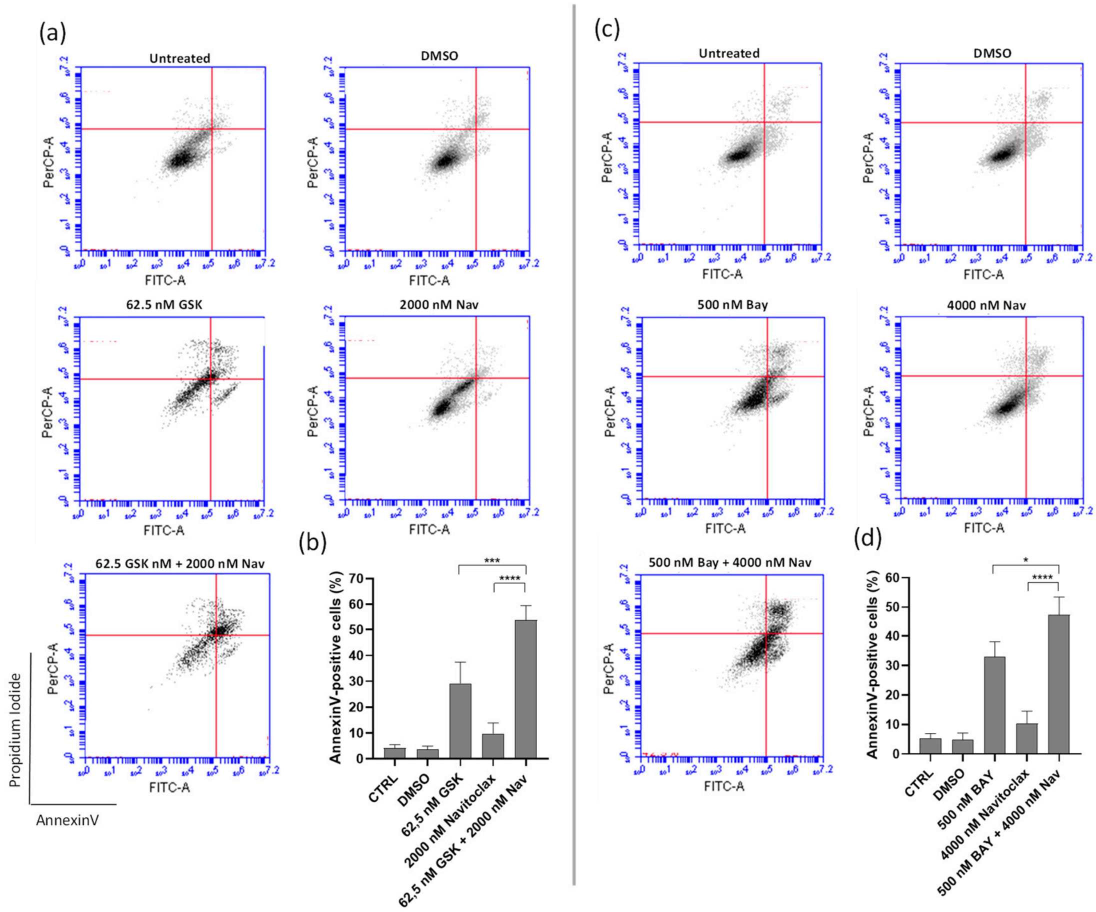

Figure S4: GSK923295 + navitoclax and BAY1217389 + navitoclax combinations enhance NCI-H460 lung cancer cell death. Representative cytograms of NCI-H460 cell line double stained with Annexin V-FITC and propidium iodide (PI) (a and c). The quadrants Q were defined as Q1 = live (Annexin V- and PI-negative), Q2 = early stage of apoptosis (Annexin V-positive/PI-negative), Q3 = late stage of apoptosis (Annexin V- and PI-positive) and Q4 = necrosis (Annexin V-negative/PI-positive). Quantification of Annexin-V-positive cells (b and d). Data represent the mean ± SD of three independent experiments, One-way ANOVA followed by Tukey's multiple comparisons test. * $p < 0.05$; *** $p < 0.001$; **** $p < 0.0001$.

1000 nM
Navitoclax.mp4

Video S1: Time-lapse imaging (DIC microscopy) of a A549 cell treated with 1000 nM of navitoclax undergoing a normal mitosis; time is shown in minutes; available online at https://youtu.be/JsgMBkAczFc (accessed on 3 November 2023).

125 nM
GSK923295.mp4

Video S2: Time-lapse imaging (DIC microscopy) of a A549 cell treated with 125 nM of GSK923295 undergoing slippage and remaining alive; time is shown in minutes; available online at https://youtu.be/g4_5Okc5OYw (accessed on 3 November 2023).

125 nM GSK923295
+ 1000 nM Navitocla›

Video S3: Time-lapse imaging (DIC microscopy) of a A549 cell treated with 1000 nM of navitoclax + 125 nM of GSK923295 undergoing death in mitosis; time is shown in minutes; available online at https://youtu.be/xi9csADTsDI (accessed on 3 November 2023).
